# Risk prediction models for oral squamous cell carcinoma and oral potentially malignant disorders in Asian populations: a systematic review and meta-analysis

**DOI:** 10.3389/froh.2026.1779476

**Published:** 2026-04-29

**Authors:** Emmanuel Kwateng Drokow, Cecilia Amponsem Boateng, Yongping Zhang, Xiaona He, Hao Wu, Yu Cao, Bin Wu, Lu Wang, Haoran Xu, Leizhen Xia, Wei Gao

**Affiliations:** 1 School of Public Health, Institute of Biomedical Innovation, Jiangxi Medical College, Nanchang University, Nanchang, China; 2 Jiangxi Provincial Key Laboratory of Disease Prevention and Public Health, Nanchang University, Nanchang, China; 3 Adventist Health Study, Research Affairs, Loma Linda University, Linda, CA, United States

**Keywords:** mouth neoplasms, oral potentially malignant disorders (OPMDs), oral squamous cell carcinoma (OSCC), predictive models, risk assessment, sensitivity

## Abstract

**Background:**

Oral squamous cell carcinoma (OSCC) and oral potentially malignant disorders (OPMDs) represent a substantial public health burden across Asia, where tobacco use, betel quid chewing, and alcohol consumption are prevalent risk factors. Numerous multivariable risk prediction models have been developed to support early detection and targeted screening; however, their accuracy and applicability in Asian populations remain unclear. This study evaluated the diagnostic performance, methodological quality, and clinical readiness of OSCC/OPMD risk prediction models developed or validated in Asian settings.

**Methods:**

Following PRISMA and PRISMA-DTA guidelines, a comprehensive search of PubMed, Embase, Cochrane Library, Scopus, Google Scholar and grey literature was conducted for studies published from January 2000 to December 2025. Eligible studies included multivariable prediction models developed or validated among asymptomatic adults in Asian countries. Data extraction and PROBAST risk-of-bias assessments were completed independently by two reviewers. Hierarchical summary receiver operating characteristic (HSROC) modelling was used to pool sensitivity and specificity, with heterogeneity explored using meta-regression. Publication bias was assessed using Deeks' test. All analyses were performed in RStudio.

**Results:**

Eleven studies met the inclusion criteria, and seven contributed to the meta-analysis. Pooled sensitivity was 0.84 (95% CI: 0.78–0.89), and pooled specificity was 0.80 (95% CI: 0.67–0.89), demonstrating strong case-detection performance with acceptable false-positive rates. Substantial heterogeneity was observed (sensitivity I^2^ = 91.4%; specificity I^2^ = 99.2%). Meta-regression showed significantly higher sensitivity among externally validated models and those predicting combined OSCC/OPMD outcomes. No significant publication bias was detected.

**Conclusion:**

Risk prediction models for OSCC and OPMDs in Asian populations demonstrate favourable diagnostic performance; however, substantial heterogeneity and methodological limitations warrant cautious interpretation before clinical implementation. Future research should prioritise large-scale external validations, improved calibration reporting, and harmonised risk thresholds to enhance regional applicability for risk-based oral cancer screening.

**Systematic Review Registration:**

https://www.crd.york.ac.uk/PROSPERO/search

## Introduction

Oral squamous cell carcinoma (OSCC) represents a significant public health burden in Asia, which accounts for a large proportion of global oral cancer incidence. In 2020, Asian countries accounted for nearly two-thirds of the 377,000 new oral cancer cases worldwide (1). Incidence rates are among the highest globally in parts of South and Southeast Asia, with some countries like India, Bangladesh, Sri Lanka reporting age-standardized rates above 12–15 per 100,000 and contributing over half of worldwide oral cancer deaths (2). This burden is associated with the high prevalence of established risk factors, including tobacco use particularly smokeless tobacco, betel quid chewing, and alcohol consumption (2). For instance, of the 360 million smokeless tobacco users globally, nearly 80% reside in Asia, and the association between these products and oral cancer is well established (2). Oral potentially malignant disorders (OPMDs), a group of oral mucosal lesions with an increased risk of malignant transformation, are also prevalent in Asian communities with high-risk habits (3).

Early detection of OSCC and OPMDs is associated with improved survival and clinical outcomes when lesions are identified at premalignant or early cancer stages (4). However, because OSCC has a relatively low prevalence in the general population, universal population-wide screening is inefficient and resource-intensive (5, 6). Risk prediction models have been developed to stratify individuals according to estimated risk and support targeted screening approaches (7). In recent decades, numerous multivariable risk models have been developed to predict an individual's risk of developing oral cancer or detecting OPMDs in order to guide risk-stratified screening programs (8). These models typically combine demographic, behavioural, and sometimes genetic or biomarker predictors to estimate risk.

Risk prediction models are multivariable tools designed to estimate disease probability within a defined time frame or to identify prevalent pathology during screening. Model performance is commonly evaluated using measures of discrimination, such as the area under the receiver operating characteristic curve (AUC), alongside sensitivity and specificity at defined risk thresholds. However, discrimination alone is insufficient; robust prediction models also require appropriate calibration, external validation in independent populations, and transparent reporting to ensure reliability and transportability. Although several oral cancer risk models have demonstrated favourable discriminatory performance (4), uncertainty remains regarding their generalisability and methodological robustness, particularly in Asian populations where risk factor profiles and healthcare systems vary considerably.

Only a limited number of models have undergone external validation in independent populations, and reported performance measures, including sensitivity, specificity, and calibration, vary across studies. Differences in study design, predictor selection, and outcome definitions may further affect model transportability across diverse Asian settings. To date, no comprehensive synthesis has quantified the diagnostic accuracy of OSCC and OPMD risk prediction models specifically within Asian populations. Therefore, we conducted a systematic review and meta-analysis to identify, appraise, and synthesise risk prediction models developed and/or validated in Asian populations for identifying individuals at risk of OSCC or OPMDs in screening contexts.

## Methods and materials

### Study protocol registration

This review was conducted according to the Preferred Reporting Items for Systematic Reviews and Meta-Analyses (PRISMA) guidelines and the PRISMA extension for diagnostic test accuracy (PRISMA-DTA) (9, 10). The study protocol was registered with the International Prospective Register of Systematic Reviews (PROSPERO) before the review commenced (CRD420251235940).

### Information sources and search strategy

We conducted a comprehensive literature search to identify all relevant studies. The following databases were searched: PubMed, Embase, and the Cochrane Library (CENTRAL). In addition, we searched Scopus or Web of Science for broader coverage of interdisciplinary journals, and Google Scholar for any difficult-to-find or grey literature. The search covered all records from January 2000 to December 2025. The search strategy was developed in consultation with an experienced medical librarian. The search incorporated both controlled vocabulary terms such as MeSH terms and free-text keywords. These terms were structured around three core concepts: oral cancer, risk prediction modelling, and Asia.

For the oral cancer component, we used terms including “oral cancer”, “mouth neoplasms”, “oral squamous cell carcinoma”, and specific OPMD-related terms such as “oral leukoplakia”, “oral potentially malignant disorder”, and “oral submucous fibrosis”. To capture studies focused on risk prediction, we applied terms such as “risk model”, “risk prediction”, “risk score”, “predictive model”, “logistic model”, “algorithm”, “machine learning”, and “neural network”. The final concept, Asia was addressed using general terms such as “Asia” and “Asian”, as well as the names of individual Asian countries. Country names were not restricted during the database searches themselves to avoid inadvertently excluding studies that did not explicitly mention the study location in the title or abstract. Instead, eligibility regarding Asian settings was confirmed during the study selection phase. The search strategy was tailored for each database. For PubMed, for example, the search combined terms such as: (“oral OR mouth”) AND (“cancer OR neoplasm” OR “potentially malignant” OR “leukoplakia” OR “submucous fibrosis”) AND (“risk OR predict” OR “model” OR “score” OR “algorithm”). All retrieved records were imported into reference management software, and duplicate entries were removed prior to screening. This systematic and multi-layered search approach ensured comprehensive coverage of the literature relevant to prediction modelling for early detection of OSCC and OPMDs in Asian populations.

### Eligibility criteria

This review included studies that developed or validated prediction models for estimating the risk of OSCC or OPMDs in Asian populations. Predefined eligibility criteria were applied to ensure the relevance and applicability of included studies.

The population of interest comprised adults aged 18 years or older residing in any Asian country or territory, spanning South Asia, East Asia, Southeast Asia, West Asia/Middle East, and Central Asia. Eligible participants were required to be asymptomatic at the time of risk assessment, with no prior diagnosis of OSCC and no current clinical signs suggestive of oral cancer. This population included community-dwelling adults, individuals attending general dental clinics or primary care facilities, and those participating in community or institutional screening initiatives. Studies involving individuals with high-risk habits, such as tobacco use or betel quid chewing, were included provided that participants were otherwise asymptomatic.

Second, studies were required to report on the development or validation of a multivariable risk prediction model. Eligible models included those designed to estimate an individual's risk of developing OSCC or an OPMD, as well as models intended to identify individuals at elevated risk for the purposes of screening or preventive interventions. Both original model development studies with or without internal validation and external validation studies of pre-existing models conducted in Asian populations were included. The review accepted models using any combination of predictors, such as demographic characteristics, lifestyle exposures, clinical oral findings, genetic markers, or serological indicators. Eligible modelling approaches included logistic regression, Cox regression, machine learning algorithms, and neural network methods. In addition, models predicting the presence of OPMDs at the point of screening were included, given that OPMDs represent established precursors of OSCC, and their early detection aligns with the overarching objective of improving oral cancer prevention.

The primary outcomes of interest were incident OSCC or the detection of OPMDs. Eligible studies reported either incident OSCC occurring during a defined follow-up period or the detection of OPMDs (leukoplakia, erythroplakia, or oral submucous fibrosis) in screening or community-based populations. Outcome determination was required to be based on a valid reference standard, typically a histopathologically confirmed diagnosis for OSCC or a clinical or histological diagnosis for OPMDs. Furthermore, studies were required to report at least one measure of predictive performance, such as sensitivity, specificity, or the area under the receiver operating characteristic curve (AUC), to allow evaluation of the model's discriminatory ability.

In terms of study design, observational study designs were eligible, including cohort studies, cross-sectional studies, and case–control studies commonly used for retrospective model development. Both prospective and retrospective designs were included. No minimum sample size threshold was imposed; however, studies were required to provide sufficient data to allow assessment of the model's performance. Studies conducted in any screening or early detection setting within Asia were considered, including community-based programmes, dental or medical clinics, hospital outpatient departments, and population-based cohorts.

Only studies published in English were included due to resource constraints associated with translation. Eligible studies were those published from January 2000 to December 2025. Peer-reviewed journal articles were the primary focus; however, grey literature was also considered if adequate data were available.

### Exclusion criteria

Several exclusion criteria were also applied. Studies conducted entirely outside Asia or involving populations not from Asian countries were excluded unless data for Asian participants could be clearly isolated. Research developing models for purposes unrelated to OSCC or OPMD risk such as survival prediction among diagnosed cancer patients or models focusing solely on oropharyngeal cancer was also excluded. In addition, studies exclusively involving individuals with established disease, such as those predicting malignant transformation among patients with known OPMDs or studies of nodal metastasis in confirmed OSCC cases, were not eligible, as they fall outside the scope of identifying risk among asymptomatic individuals. Studies that did not develop or validate a multivariable risk prediction model, such as those reporting only univariable analyses or descriptive associations, were excluded. Non-primary research articles, including reviews, commentaries, editorials, letters, and case reports, were also excluded, although reference lists of relevant reviews were screened for potentially eligible primary studies. When multiple publications were identified from the same dataset, the most comprehensive report was included, with supplementary publications used only for additional contextual information.

### Study selection

Study selection followed a two-stage process consistent with PRISMA guidelines. In the first stage, two reviewers independently screened the titles and abstracts of all retrieved records to identify potential eligibility. A liberal inclusion approach was applied, whereby any record judged as potentially relevant by either reviewer was carried forward to full-text review. In the second stage, full-text articles of all candidate studies were obtained and evaluated in detail by two independent reviewers against the predefined eligibility criteria. A standardised inclusion and exclusion form was used to ensure consistency across reviewers. Any disagreements at either stage were resolved through discussion and consensus, with a third reviewer consulted when necessary. Reasons for exclusion at the full-text stage were documented. The overall selection process was summarised in a PRISMA flow diagram, detailing the number of records identified, screened, included, and excluded at each step.

### Data extraction

Data extraction was conducted using a structured, piloted extraction form developed for this review. Two reviewers independently extracted data from each included study, with discrepancies resolved through discussion or consultation with a third reviewer. The extraction form was piloted on a small sample of studies and refined before full data abstraction to ensure clarity, completeness, and uniform application. From each included study, we extracted general study information, including authorship, year of publication, and country of study. Study design, setting, and type of recruitment setting were also recorded. The population characteristics were documented, including total sample size for development and/or validation cohorts. For validation studies, we recorded whether the sample represented an internal or external validation.

Detailed information on each prediction model was extracted. Extracted variables included modelling approach, predictors included in the final model, and methods used to measure these predictors. Outcome definitions and ascertainment methods were captured, including whether the study assessed incident OSCC during follow-up period, prevalent OPMDs identified at screening, or combined outcomes. All reported performance metrics were extracted. These included measures of discrimination such as the AUC or C-statistic with confidence intervals where available, as well as sensitivity, specificity, and predictive values at reported risk thresholds. When studies defined a high-risk cutoff or action threshold, the corresponding diagnostic performance metrics were also recorded. When critical information was missing or unclear, study authors were contacted for clarification. Authors were given at least 1–2 weeks to respond before analyses proceeded with the available data.

### Quality assessment of studies

The methodological quality and risk of bias of each included study were assessed independently by two reviewers using the PROBAST tool (Prediction model Risk Of Bias Assessment Tool) (11). PROBAST is specifically designed to evaluate prediction model studies and assesses risk of bias across four domains: Participants, Predictors, Outcome, and Analysis (11). For each study, all PROBAST signalling questions were completed to inform domain-specific judgments of risk of bias (low, high, or unclear). Concerns about applicability such as whether a study's population, predictor set, or outcome definition aligned with the aims of this review were also documented. Any disagreements between reviewers were resolved through discussion, with consultation of a third reviewer when necessary. The results of the quality assessment were summarised in structured tables and graphical outputs to illustrate patterns of bias across studies. Common methodological limitations identified included potential selection bias in clinic- or hospital-based samples, inconsistent or poorly described measurement of predictors, lack of explicit blinding of outcome assessment, inadequate handling of missing data, and risks of overfitting due to small sample sizes or limited validation procedures. These limitations were considered when interpreting model performance and overall study reliability.

### Statistical analysis

Both quantitative and qualitative synthesis methods were undertaken in this review. Descriptive analyses were first conducted to summarise key study characteristics, population features, modelling approaches, predictors, and outcome definitions. Results were presented in narrative text, tables, and summary figures. All included studies were eligible for qualitative synthesis; however, only studies reporting sufficient information to derive sensitivity and specificity were included in the quantitative meta-analysis. For studies that did not directly report sensitivity or specificity, these metrics were calculated, where possible, from contingency tables or other reported performance estimates. Forest plots were generated to display the sensitivity, and specificity estimates for individual models across studies.

Because the included models applied varying probability thresholds to classify individuals as high risk, pooled estimates of sensitivity and specificity were synthesised using a random-effects hierarchical summary receiver operating characteristic (HSROC) model. The HSROC approach jointly models sensitivity and specificity while accounting for threshold effects and between-study heterogeneity, making it appropriate when diagnostic performance metrics are reported at different decision cut-offs. Although most commonly used in diagnostic test accuracy meta-analyses, the HSROC framework is equally applicable to risk prediction models when their performance is evaluated using binary classification measures in screening contexts. Summary points, along with 95% confidence and prediction regions, were derived from the fitted HSROC curve.

Statistical heterogeneity in pooled estimates was assessed using Cochran's *Q* test and Higgins' I² statistic. A *p*-value <0.10 for Q and I² values above 50% were interpreted as evidence of significant heterogeneity. To explore sources of heterogeneity, random-effects meta-regression analyses were conducted using the restricted maximum likelihood (REML) estimator. Prespecified covariates included model type, study population characteristics, validation method (internal vs. external), and target outcome (OSCC vs. OPMD).

Small-study effects and potential publication bias were assessed using Deeks' funnel plot asymmetry test, which is recommended for diagnostic accuracy meta-analyses and is more robust than traditional funnel plot approaches for evaluating discriminatory performance metrics (12). Sensitivity analyses were conducted to evaluate the robustness of pooled results. These included exclusion of studies judged at high risk of bias based on PROBAST, exclusion of models without external validation, and subgroup analyses restricted to homogeneous outcome types or similar modelling approaches. All statistical analyses were conducted using R statistical software (Version 2025.09.2 + 418) within the RStudio environment. The “mada”, “metafor”, “ggplot2”, “dplyr”, “tidyr” and meta packages were used for diagnostic accuracy modelling and meta-analytic procedures. These analytical approaches enabled a rigorous synthesis of the predictive performance of OSCC and OPMD risk prediction models.

## Results

### Study selection and screening

**Figure 1:**
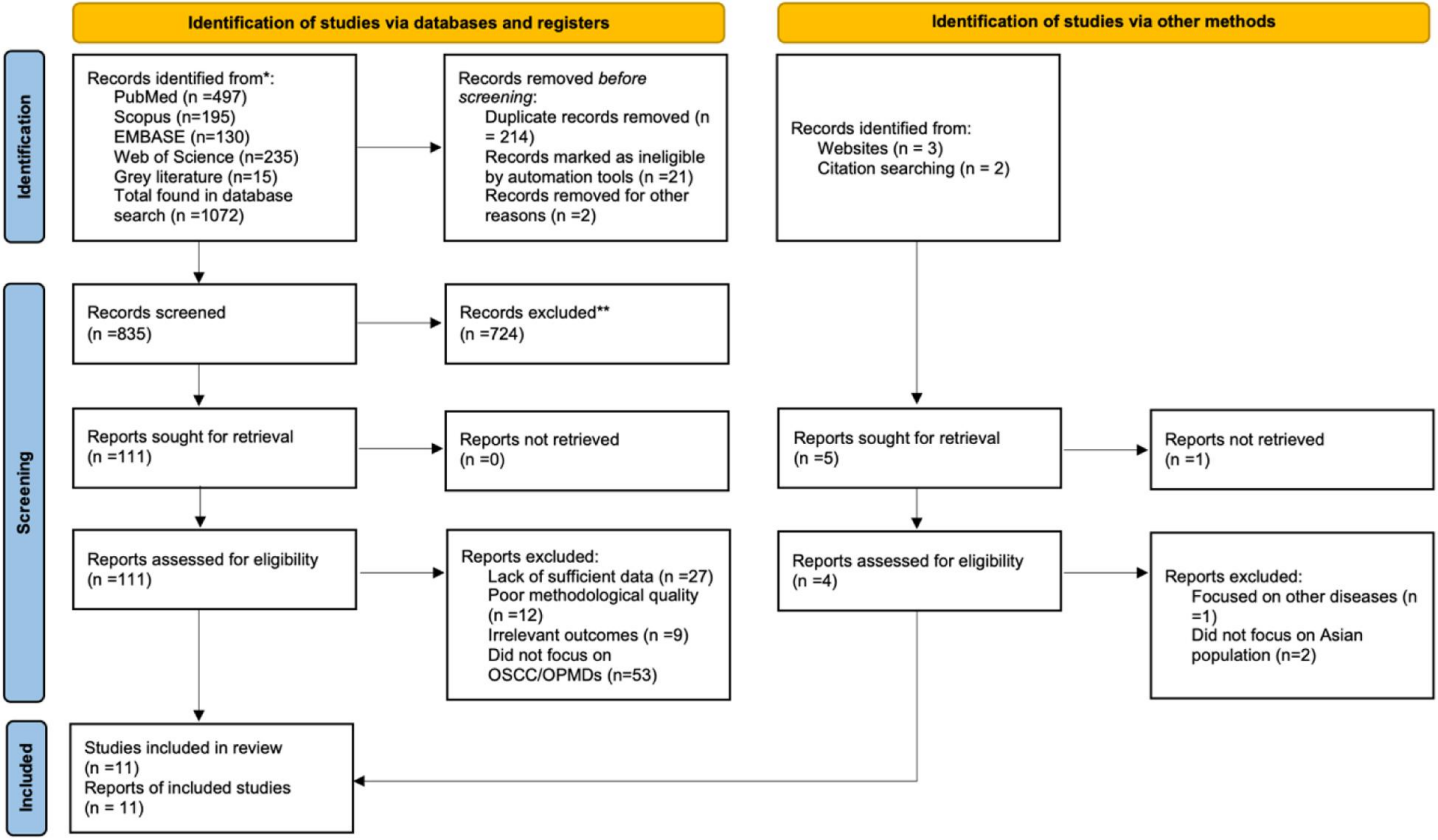
Selection of studies.

Our comprehensive database searches identified 1,072 unique records. After removal of 214 duplicates and 23 records flagged as ineligible by automation tools, 835 titles and abstracts were screened. Of these, 724 records were excluded at the title and abstract stage due to irrelevant population, outcome, or study design, leaving 111 articles for full-text review. Full-text evaluation resulted in exclusion of 100 studies, primarily because they did not report a multivariable model, involved non-Asian populations, or focused on ineligible outcomes. Ultimately, 11 studies met the inclusion criteria and were included in the qualitative synthesis. Of these, 7 studies provided sufficient diagnostic accuracy data for inclusion in the meta-analysis. The study selection process is summarized in the PRISMA flow diagram (Figure 1) (13–22). Figure 1 illustrates the number of records identified, screened, excluded at each stage, and included in the qualitative and quantitative synthesis.

### Characteristics of included studies

The 11 included studies were conducted in various regions across Asia. Three were based in China, three in Taiwan, two in India, and one each in Sri Lanka, Malaysia, and Hong Kong. Sample sizes varied widely, ranging from 171 to 1,719,191 participants (median −2,824), with reported outcome events ranging from 44 to 6,275. Nine of the eleven studies used a prospective cohort design, while two employed retrospective designs (Table 1).

**Table 1:** Selected studies characteristics.

Author & year	Type of study	Country/Region	Sample size	Type of setting	Outcome
Chiu et al., (28)	Prospective	Taiwan	217	Community	OPMDs
Amarasinghe et al. (14)	Prospective	Sri Lanka	1,179	Community	OSCC, OPMDs
Dom et al. (29)	Retrospective	Malaysia	171	Single-centre	OSCC
Krishna et al. (17)	Prospective	India	452	Multi-centre	OSCC
Miao et al. (22)	Prospective	China	2,014	Multi-centre	OSCC
Chung et al. (19)	Prospective	Taiwan	1,027	Dual-centre	OSCC
Chen et al. (15)	Prospective	China	3,624	Single-centre	OSCC
Hung et al. (18)	Retrospective	Taiwan	17,19,191	Community	OSCC
Chen et al. (15)	Prospective	China	973	Single-centre	OSCC
Cheung et al. (25)	Prospective	India	1,91,870	Community	OSCC
Adeoye et al. (13)	Prospective	Hong Kong	979	Community	OPMDs

OSCC, Oral Squamous Cell Carcinoma; OPMDs, Oral Potentially Malignant Disorders.

Most models were developed using regression techniques. Eight studies developed risk models using variants of logistic regression (including binary or multivariate logistic models) (Table 2). One study employed Cox proportional hazards regression, and two used alternative machine-learning approaches (a “fuzzy” linear regression and a multifactor dimensionality reduction model). The outcomes modelled were predominantly incident OSCC: eight studies predicted OSCC risk, two focused on OPMD detection, and one model evaluated combined OSCC/OPMD outcomes.

**Table 2:** Selected variables for the included meta-analysis study.

Author & year	Model_type	Validation type	Number of Predictors	Sensitivity	Specificity	AUCs
Chiu et al., (28)	Binary logistic regression	Internal	6	0.82	0.86	0.90
Amarasinghe et al. (14)	Multiple logistic regression	External	5	0.95	0.76	0.87
Krishna et al. (17)	Multiple logistic regression	Internal	9	0.93	0.60	0.87
Chung et al. (19)	Logistic regression	External	5	0.86	0.86	0.88
Chen et al. (15)	Multiple logistic regression	Internal	1	0.70	0.91	-
Hung et al. (18)	Multiple logistic regression	Internal	12	0.77	0.56	0.73
Adeoye et al. (13)	Multiple logistic regression	External	12	0.71	0.78	0.82

AUC, Area Under the Curve.

Regarding validation, eight models reported internal validation, typically using cross-validation or split-sample techniques, whereas three studies reported external validation in independent datasets. The predictors included in the models spanned multiple domains. All models incorporated basic demographic factors typically age and sex, and nearly all included behavioural/lifestyle variables such as tobacco use, alcohol consumption, or betel quid chewing. Many models also included clinical oral variables for example, the presence or characteristics of oral lesions or OPMDs, oral hygiene indicators, or body mass index. A minority of models incorporated molecular or serological biomarkers for example, a few models included viral infection markers or other blood-based biomarkers. Overall, the included studies used a diverse range of predictors (demographic, behavioural, clinical, and, in some cases, biological) to develop multivariable risk models for OSCC and OPMD in Asian populations.

### Meta-analysis of model performance

**Figure 2:**
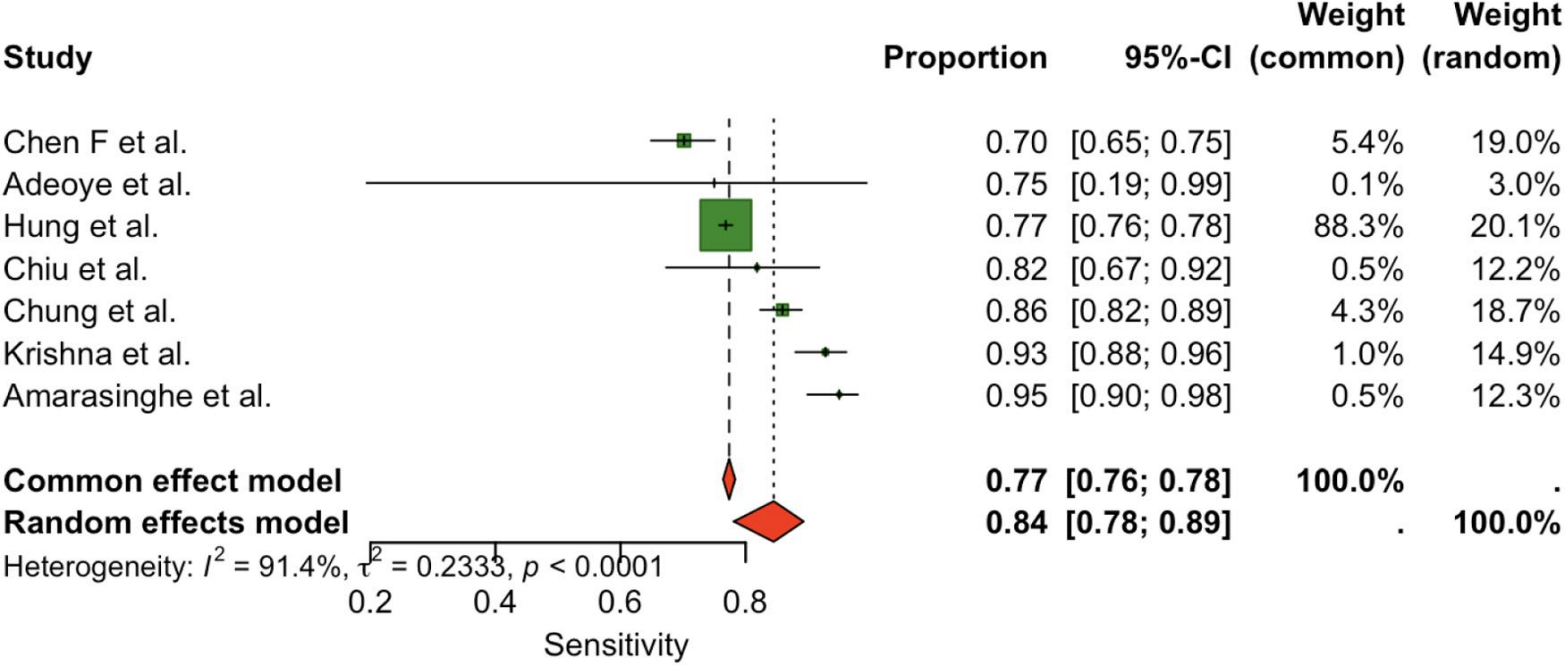
Pooled sensitivity for the studies.

**Figure 3:**
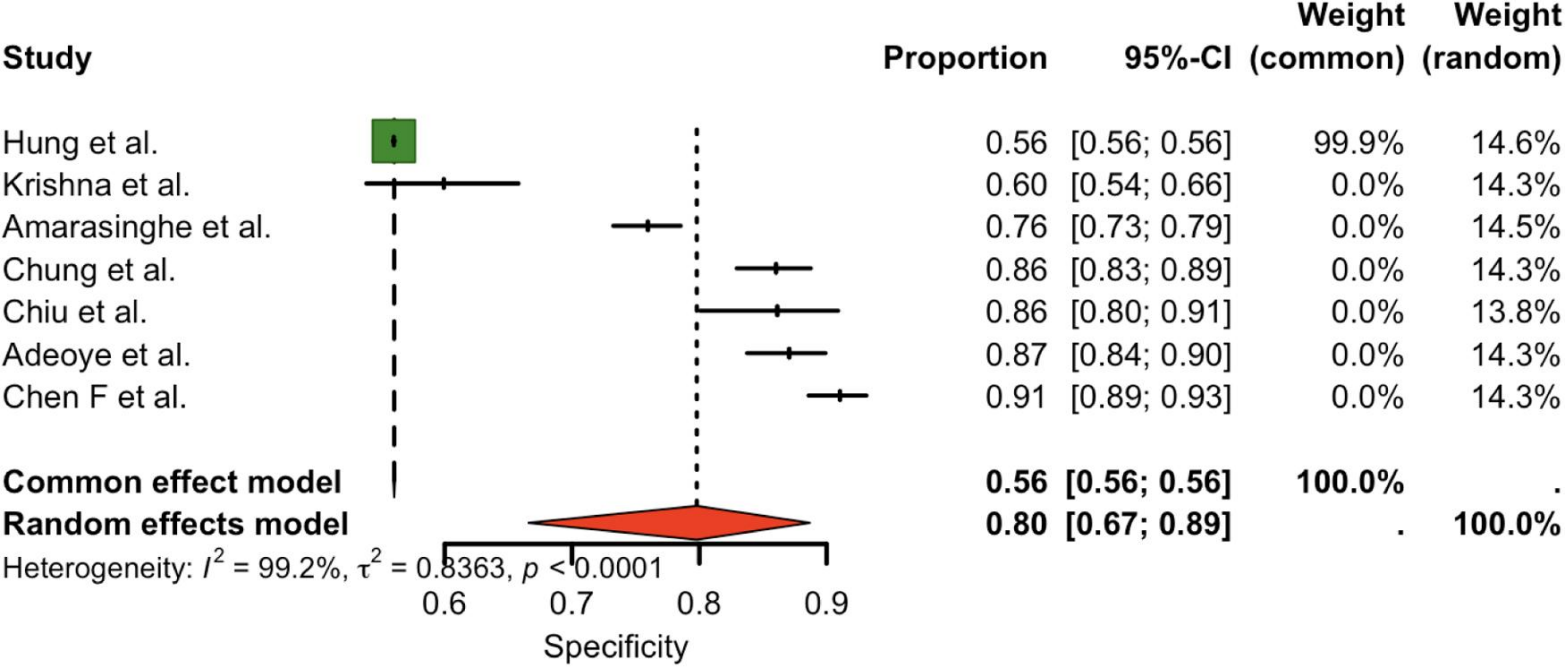
Pooled specificity for the studies.

Seven studies (total *n* = 7) were included in the meta-analysis. The pooled sensitivity of the OSCC/OPMD risk prediction models was (0.84, 95% CI: 0.78–0.89), indicating strong case-detection performance. Figure 2 presents the forest plot of sensitivity estimates across included studies, demonstrating consistently high sensitivity. Pooled specificity was (0.80, 95% CI: 0.67–0.89). Figure 3 displays the forest plot of specificity estimates across studies, showing greater variability. However, there was substantial between-study heterogeneity in both metrics: for sensitivity, I² = 91.4% (*τ*² = 0.2333, *p* < 0.0001); for specificity, I² = 99.2% (*τ*² = 0.8363, *p* < 0.0001). These high I² values indicate that the variation in reported accuracy exceeded that expected by chance.

**Figure 4:**
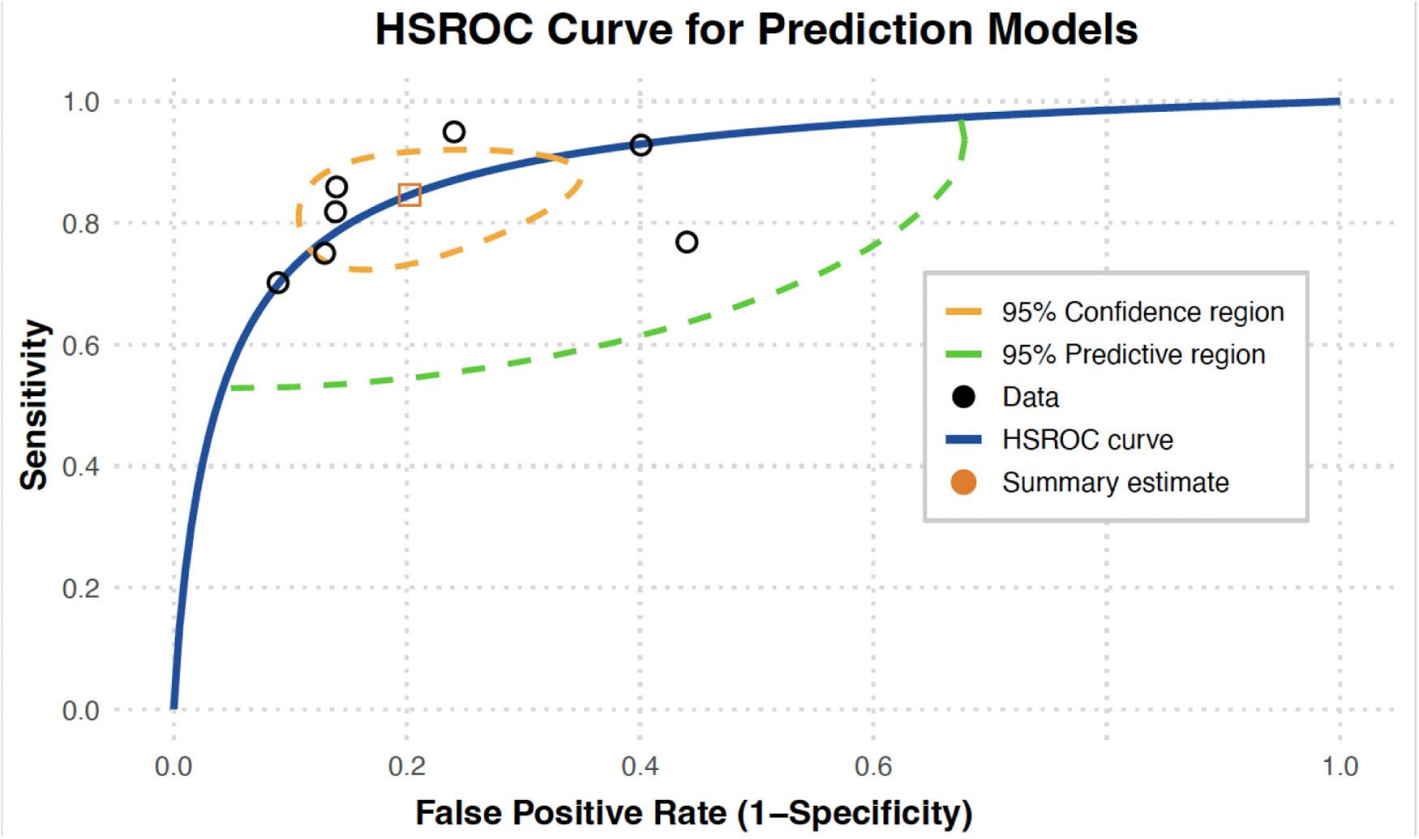
Pooled ROC for the included studies.

**Figure 5:**
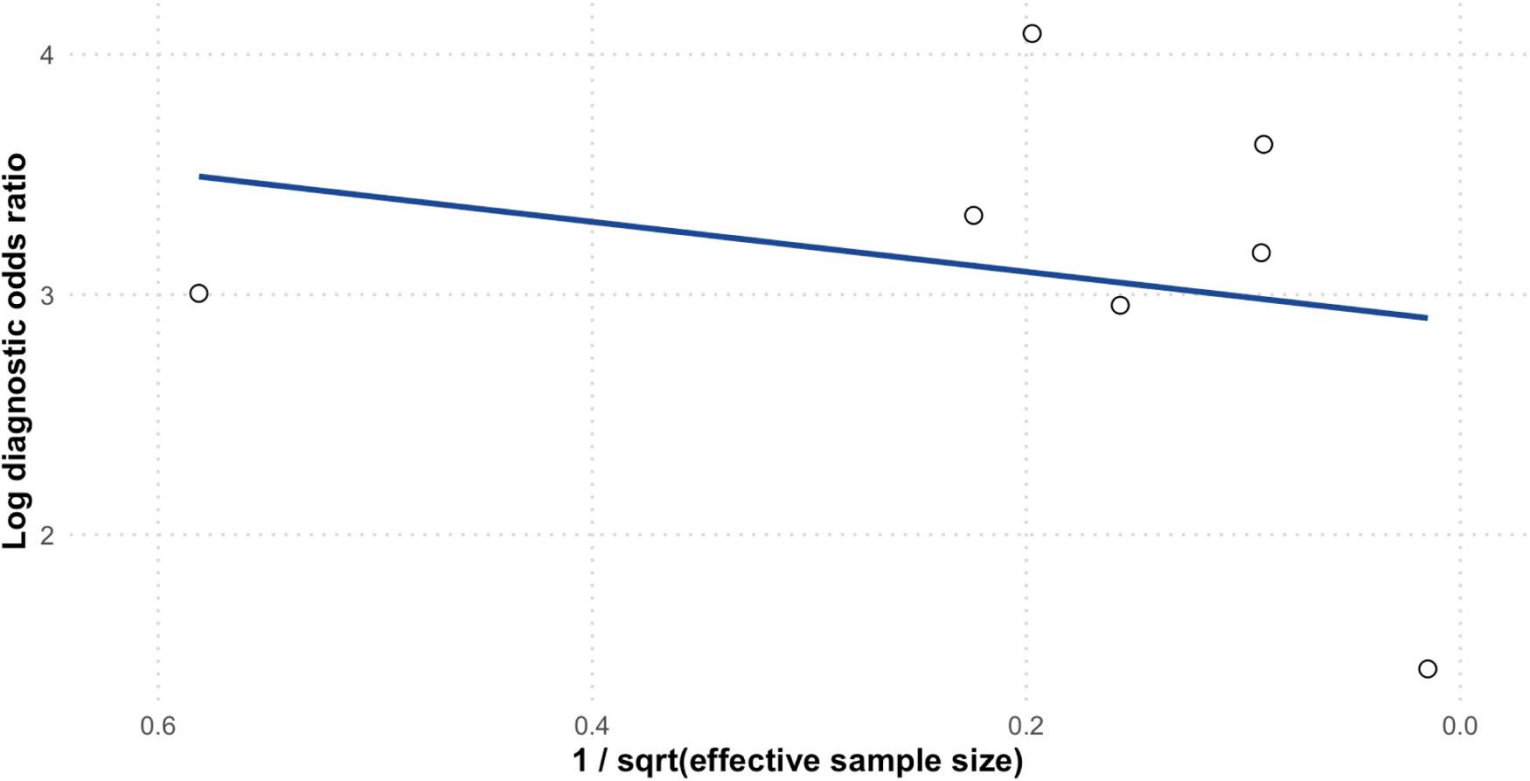
Funnel plot.

The HSROC summary point was located near the upper-left region of the plot (high sensitivity and low false-positive rate), with 95% confidence and prediction regions indicating relatively consistent performance across studies. Figure 4 presents the HSROC curve, illustrating the pooled summary point along with 95% confidence and prediction regions. Deeks' funnel plot did not show significant asymmetry, suggesting a low likelihood of publication bias (Figure 5). Nonetheless, the observed heterogeneity likely arose from differences in study and model characteristics. For instance, the included models varied in predictor sets, case definitions, diagnostic thresholds, and population demographics. These variations in design and patient mix likely contributed to the wide heterogeneity in sensitivity and specificity observed.

These findings suggest that the pooled models demonstrate favourable performance characteristics for screening applications. High sensitivity is especially desirable for early detection of OSCC/OPMD, and the pooled sensitivity of 0.84 implies that most true cases would be correctly identified by these models. The moderate specificity (0.80) means there would be some false positives, which is generally acceptable in a screening context where minimizing missed cases is a priority. Overall, the high sensitivity and favourable HSROC performance indicate that these risk prediction models may effectively identify high-risk individuals in Asian populations. However, the considerable heterogeneity cautions that performance may vary in different settings, underscoring the need for careful calibration or validation before implementation.

### Meta-regression findings

The meta-regression revealed statistically significant effects for both validation type and target condition on diagnostic sensitivity. Prediction models that underwent external validation demonstrated significantly higher sensitivity than those evaluated using internal validation only (logit estimate = 0.982; *p* = 0.018) (Table 3). This finding suggests that externally validated models may demonstrate improved generalisability and reduced overfitting compared with internally validated models. These results indicate that externally validated models may maintain stronger diagnostic accuracy when applied beyond their development datasets.

**Table 3:** Meta-regression to determine heterogeneity.

Variable	Study numbers	Sensitivity Estimate	Sensitivity *p*-value	False Positive Rate Estimate	FPR *p*-value
Validation type					
Internal (ref)	4	—	—	—	—
External^ᵃ^	3	0.982	0.018	−0.421	0.301
Target condition					
OSCC (ref)	4	—	—	—	—
OPMDs/OSCC^ᵃ^	3	1.217	0.009	−0.503	0.275

ᵃ Reference category in the meta-regression model (its coefficient is set to 0, so estimates are differences for the other level vs. this reference). OSCC, Oral Squamous Cell Carcinoma; OPMDs, Oral Potentially Malignant Disorders.

A similar significant effect was observed for target condition. Models that combined OPMDs and OSCC outcomes exhibited significantly higher sensitivity compared with those designed solely to detect OSCC (logit estimate = 1.217; *p* = 0.009). This positive association may reflect the broader and more diverse range of clinical features incorporated into combined-disease models, improving their ability to detect malignant or potentially malignant lesions at earlier stages. The improved sensitivity suggests that integrating OPMDs into prediction frameworks may enhance early detection performance across the disease continuum.

In contrast, neither moderator produced statistically significant effects on false-positive rates, indicating that gains in sensitivity did not come at the cost of reduced specificity. This separation of effects strengthens the conclusion that variations in sensitivity are likely attributable to meaningful study-level characteristics rather than artefacts of measurement or bias. Overall, the significant findings provide compelling evidence that external validation and combined OPMDs/OSCC target conditions are associated with superior diagnostic sensitivity, highlighting these factors as potential contributors to improved model performance and generalisability in clinical settings.

### Quality assessment (risk of bias)

**Figure 6:**
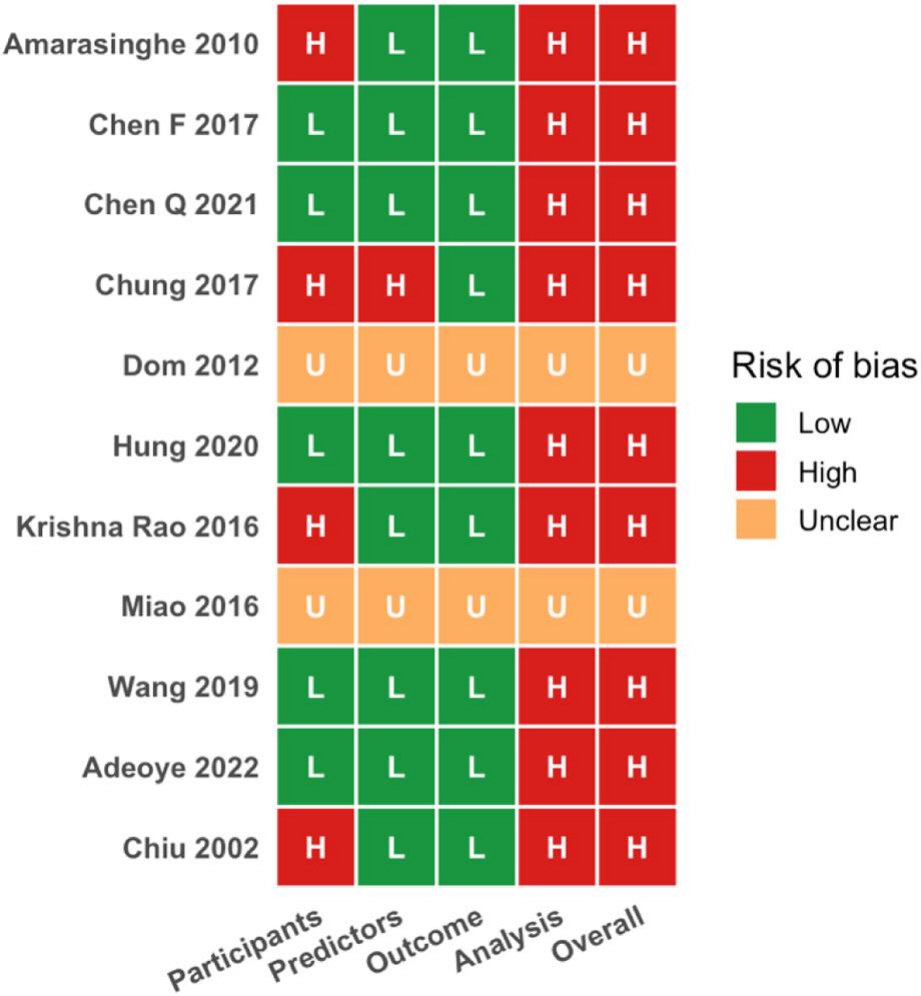
PROBAST risk-of-bias judgments by domain for each studies.

**Figure 7:**
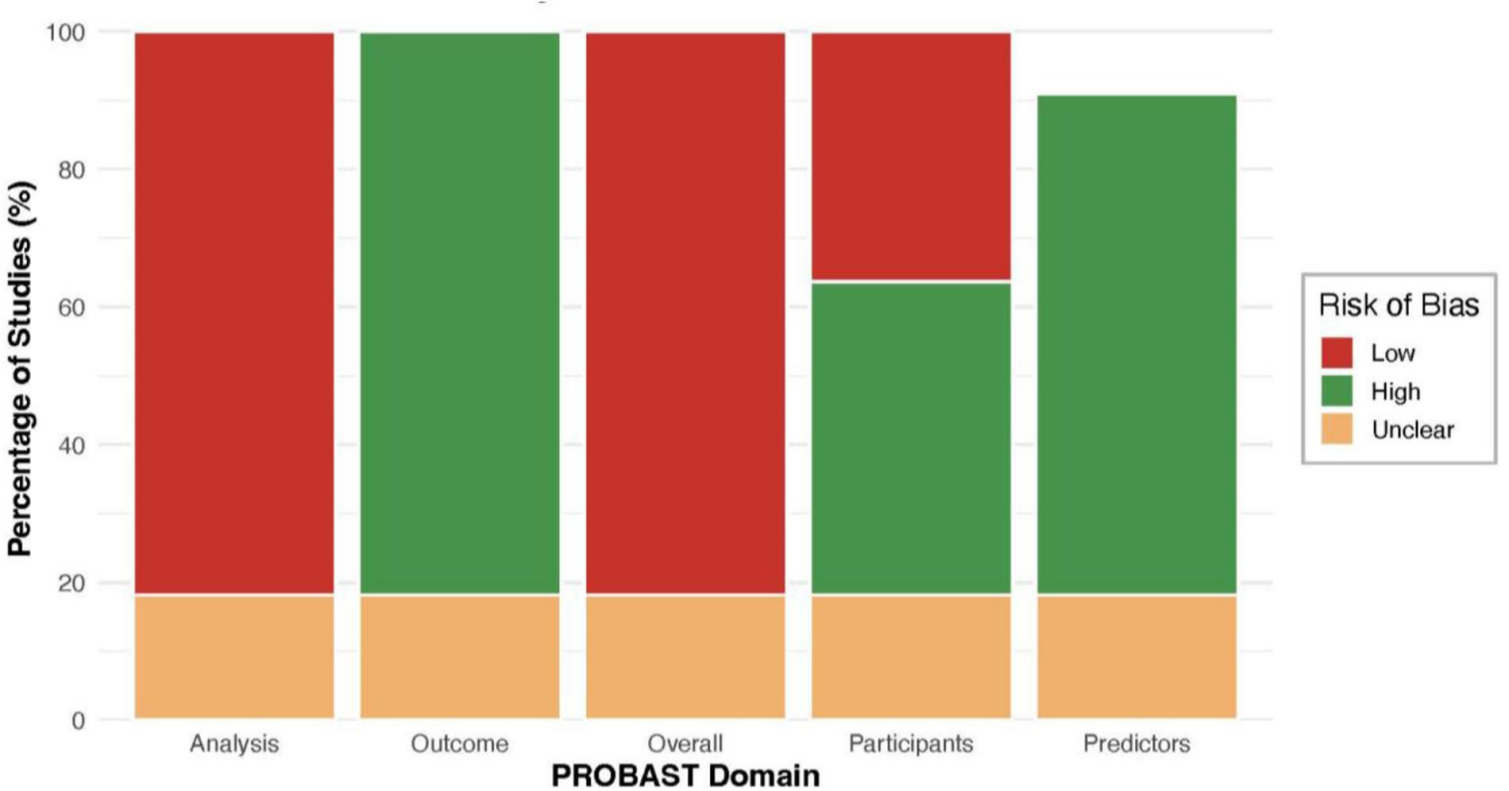
Proportion of studies with risk of bias in each PROBAST domain.

The PROBAST assessment of risk of bias (Figure 6) showed that most studies were judged to have low risk in the Participants and Predictors domains, whereas the Analysis domain was frequently rated as high risk. Figure 6 illustrates domain-level risk-of-bias assessments across included studies according to PROBAST criteria. A few studies also had high or unclear risk in the Outcome domain. In total, roughly half of the studies were classified as high risk of bias overall (Figure 7). Figure 7 summarises the proportion of studies classified as low, high, or unclear risk of bias across each PROBAST domain. Common drivers of high-risk judgments were found in the analysis domain. Many models had small sample sizes and lacked rigorous validation (often using only the development data), raising concerns about overfitting. Reporting of key analyses was often incomplete for example, calibration statistics were rarely provided and methods for handling missing data were poorly described. These and related issues such as selection bias in convenience samples and lack of blinding in outcome assessment were frequently noted during quality assessment. Therefore, inadequate validation procedures, overfitting due to limited sample size, and incomplete reporting (calibration and missing data) were recurrent concerns in the high-risk studies. Applicability to the target clinical context was generally acceptable. Most prediction models were developed in populations and using predictors that matched our inclusion criteria. Only a few studies raised minor applicability concerns like, models developed exclusively in highly selected hospital cohorts might not be fully generalizable to broader screening populations. Overall, no major misalignment in predictor selection or outcome definitions was observed.

## Discussion

This systematic review and meta-analysis demonstrated that multivariable risk prediction models for OSCC and OPMDs in Asian populations show substantial diagnostic accuracy. The pooled sensitivity (0.84) and specificity (0.80) indicate that these models identify a high proportion of true cases while excluding most low-risk individuals. These performance estimates suggest suitability for early detection, as high sensitivity supports case identification while moderate specificity limits excessive false-positive referrals. This balance favours sensitivity, which is desirable in screening contexts, while maintaining acceptable false-positive rates. The discriminative performance observed is comparable to risk prediction tools developed for other cancers, which commonly report area-under-the-curve values between 0.70 and 0.80 (23, 24). These findings indicate that oral cancer risk models demonstrate performance comparable to models used in other oncologic contexts.

Despite the generally good aggregate performance, we observed significant heterogeneity in model outcomes across studies. Statistical heterogeneity was high, indicating considerable variation in accuracy across models and settings. This variability likely stems from multiple sources. The included studies differed in their populations, in the predictors used, and in outcome definitions. Differences in study design and patient characteristics may contribute to variation in reported performance metrics. Our meta-regression provided some insight into the heterogeneity. We found that models which had undergone external validation on independent data sets showed significantly higher sensitivity than those only assessed with internal or developmental samples. Externally validated models showed improved case-detection, potentially reflecting reduced overfitting and improved generalisability. Likewise, models aimed at predicting a combined outcome either an OPMD or OSCC occurrence were significantly more sensitive than models focused solely on OSCC detection. Inclusion of OPMDs in the outcome definition may increase sensitivity by capturing individuals with earlier pathological changes. Importantly, improvements in sensitivity were not accompanied by statistically significant reductions in specificity. This suggests that the improved sensitivity is a real enhancement in identifying true at-risk cases rather than an artifact of a lowered threshold. Thus, heterogeneity is a concern, but it can be partially explained. Models generalised to new populations and those encompassing the full continuum of oral malignant potential tend to perform better in terms of detection, highlighting key avenues for improving model design.

Our findings both corroborate and extend prior literature on oral cancer risk models. Espressivo et al. (4) conducted a global review of oral cancer risk prediction models and noted similarly that many published models showed good discriminatory power, with most reporting AUROC values above 0.70 (4). They also emphasized, however, that very few models had been validated outside of their development datasets. This lack of validation aligns with our observations in the Asian context. Among the studies we included, only three performed an external validation using a separate population, and these were the models that achieved higher sensitivities on average. The dearth of external validation is a critical gap also highlighted by previous studies, who cautioned that the heterogeneity of populations and risk factors makes it difficult to compare models or assume transferability without such testing (4). By quantitatively pooling the available data, our review adds an aggregate estimate of accuracy specific to Asian settings something previous narrative reviews could not provide. We found an overall sensitivity that indicates strong case-finding ability across models, but we echo the sentiment of prior researchers that no single model can yet be endorsed for routine clinical use in Asia without further evidence. Thus, the general agreement between our conclusions and other previous studies lends weight to a consistent message that is numerous risk models for oral cancer exist and show promise, but their clinical utility remains hampered by methodological limitations and context-specific uncertainties. Our work reinforces this message with concrete data from Asia, where the burden of disease is high and such tools are arguably most needed.

An important finding of this review concerns the methodological quality and risk of bias of the included studies. Using the PROBAST tool, we found that approximately half of the included studies were rated at high risk of bias overall. The Analysis domain was most frequently rated as high risk of bias. We observed that the majority of studies had a high risk of bias in the analysis phase. Common issues included overfitting, lack of reporting on model calibration, and incomplete handling of missing data. Furthermore, very few studies reported calibration statistics or plots, which are essential for understanding whether the predicted absolute risks are reliable. Many models were evaluated only within development datasets or using internal resampling techniques, which may produce optimistic performance estimates. Such practices may overestimate model performance, with reduced accuracy when applied to external populations. We also noted that some studies did not clearly blind outcome assessment or used cross-sectional designs for detecting OPMDs, raising potential bias in the Outcome domain. Nevertheless, on the positive side, most studies used appropriate participant inclusion and predictor measurement, meaning the populations and risk factors generally matched our review's intent. Overall, however, the presence of considerable bias risk means our pooled estimates of accuracy should be interpreted with caution. Therefore, while we have demonstrated that accurate risk tools are achievable, we must temper enthusiasm by recognizing that many existing models are not yet robust enough for immediate deployment.

### Implications for screening practice and policy in Asia

These findings have implications for oral cancer screening strategies in high-risk regions. The concept of using risk prediction models to triage individuals for screening is strongly supported by our finding of high sensitivity. These models may function as pre-screening tools to identify individuals most likely to benefit from further examination. In resource-constrained settings, such targeted approaches may improve screening efficiency. Evidence from the Indian subcontinent has already shown that focusing screening efforts on high-risk groups yields better outcomes and is more cost-effective than indiscriminate population-wide screening (25, 26). The re-analysis of the Kerala oral cancer screening trial demonstrated that restricting screening to the top risk strata retained most of the mortality reduction benefit of screening the entire population, while dramatically reducing the number of screenings needed (25). Similarly, economic evaluations indicate that high-risk screening is more cost-efficient, with lower cost per life-year saved, compared to mass screening of all adults (26). These findings align with our review's suggestion that risk models which typically incorporate known risk factors can identify the very individuals who concentrate the majority of risk. Policymakers in Asia are beginning to take notice: Taiwan's national oral cancer screening program is a notable example, where since 1999 the government provides free biennial oral mucosal exams to adults with high-risk habits (27). This targeted program has been associated with shifts toward earlier detection and reductions in oral cancer mortality in the Taiwanese population (27). Our findings lend support to such strategies. They suggest that if robustly validated risk models were integrated into screening programs, health authorities could refine the identification of “high-risk” individuals beyond simple habit criteria, possibly including other demographic or clinical risk indicators to capture those at elevated risk who might otherwise be missed. Hence, risk-based screening supported by validated prediction models may represent a feasible strategy in Asian settings, where the burden of oral cancer is high but resources for screening and early detection are limited. It offers a way to improve the yield of screening and could be an integral component of future oral cancer control policies across the region (25).

Again, translating these models into practice will require addressing a number of practical and contextual challenges. Asia is a vast and heterogeneous continent; risk factor patterns vary between and even within countries. For example, betel quid chewing is a predominant risk in South Asia and parts of Southeast Asia, whereas in other regions like the Middle East or Japan it is less common, and factors like HPV-related oropharyngeal cancer may play a slightly bigger role. A model developed in one population may not directly generalize to another without recalibration. We observed a minor applicability concern in some studies as models derived from specialized tertiary care settings might not perform as well in a general community screening context. This underlines that local validation is crucial. Each model should ideally be tested and if necessary, adjusted in the specific population where it is intended to be used. Another consideration is the feasibility of obtaining predictor information. All models in our review included basic demographics and lifestyle habits, which are relatively easy to assess via questionnaires or existing medical records. Many models also incorporated simple clinical oral examinations, which implies that a combined approach of questionnaire plus oral exam could be the most effective screening triage. However, a few models required more complex inputs such as genetic markers or HPV status. While incorporating biomarkers like HPV serology can improve predictive power, these additions entail higher costs and logistical demands something that must be justified in low- and middle-income regions of Asia where resources are limited. Indeed, prior research has noted that models using only readily available risk factors often perform nearly as well as those that include extensive lifestyle or biological data. This suggests a pragmatic approach for implementation: start with simpler risk models that are practical for large-scale use and only incorporate extra variables like biomarkers if there is clear evidence of a substantial gain in performance that outweighs the cost. Finally, we must consider that no matter how good a model's statistics are, its impact will depend on how it is used in the field. Integration of risk assessment into routine practice might involve training primary healthcare workers or dentists to use risk scoring tools and establishing referral pathways for high-risk individuals. To date, few if any studies have evaluated prospectively what happens when a risk model is deployed in a screening program (does it improve detection rate or reduce advanced cancer incidence over time?). Such implementation research is needed to confirm that the promising accuracy of these models actually translates into better patient outcome.

## Limitations

As with any systematic review, our study has several limitations that should be considered. Firstly, the relatively small sample of studies may not capture the full spectrum of existing risk models. Although extensive literature searches were conducted, relevant unpublished studies or non-English publications may have been missed. Oral cancer research from some Asian countries might be reported in local journals not indexed in the databases we searched. We also note that our search was concluded at the end of 2025; any very recent model studies published after our search window would not be included. Additionally, the relatively small number of included studies may have limited the statistical power of subgroup and meta-regression analyses. Furthermore, only seven studies contributed to the quantitative synthesis of diagnostic accuracy, which may have limited the precision of pooled sensitivity and specificity estimates. Although hierarchical modelling approaches such as HSROC can be applied in meta-analyses with a modest number of studies, limited sample size reduces the robustness of subgroup comparisons and meta-regression analyses.

Substantial statistical heterogeneity was observed, with I² values exceeding 90% for both sensitivity and specificity. Such heterogeneity is common in meta-analyses of prediction models and likely reflects differences in study populations, predictor variables, modelling approaches, validation strategies (internal vs. external), outcome definitions, and probability thresholds used to define high-risk status. Variation in screening settings and regional risk factor prevalence across Asian populations may have further contributed to this variability. Although the HSROC model accounts for threshold effects and between-study heterogeneity, the high I² values indicate that pooled estimates should be interpreted as average performance across diverse contexts rather than universally applicable benchmarks. Thus, our findings regarding moderators of performance should be seen as exploratory. Overall, the strength of evidence remains moderate due to methodological limitations across included studies.

## Conclusion

Risk prediction models for oral squamous cell carcinoma and oral potentially malignant disorders in Asian populations demonstrate favourable diagnostic accuracy based on pooled estimates. The meta-analysis suggests relatively high sensitivity, alongside moderately high specificity. However, substantial heterogeneity across studies and variability in model development and validation approaches warrant cautious interpretation of these findings. Differences in study populations, predictor selection, modelling techniques, and probability thresholds likely contributed to the observed variability in performance. While risk prediction models may support more efficient, risk-based approaches to oral cancer screening, further external validation, improved calibration reporting, and methodological standardisation are required before routine implementation can be recommended.

## Data Availability

The original contributions presented in the study are included in the article/Supplementary Material, further inquiries can be directed to the corresponding author/s.
